# Microvesicles as Potential Ovarian Cancer Biomarkers

**DOI:** 10.1155/2013/703048

**Published:** 2013-01-08

**Authors:** Ilaria Giusti, Sandra D'Ascenzo, Vincenza Dolo

**Affiliations:** Department of Life, Health and Environmental Sciences, University of L'Aquila, 67100 L'Aquila, Italy

## Abstract

Although the incidence of ovarian cancer is low (i.e., less than 5% in European countries), it is the most lethal gynecologic malignancy and typically has a poor prognosis. To ensure optimal survival, it is important to diagnose this condition when the pathology is confined to the ovary. However, this is difficult to achieve because the first specific symptoms appear only during advanced disease stages. To date, the biomarker mainly used for the diagnosis and prognosis of ovarian cancer is CA125; however, this marker has a low sensitivity and specificity and is associated with several other physiological and pathological conditions. No other serum ovarian cancer markers appear to be able to replace or complement CA125, and the current challenge is therefore to identify novel markers for the early diagnosis of this disease. For this purpose, studies have focused on the microvesicles (MVs) released from tumor cells. MVs may represent an ideal biomarker because they can be easily isolated from blood, and they have particular features (mainly regarding microRNA profiles) that strongly correlate with ovarian cancer stage and may be effective for early diagnosis.

## 1. Introduction

For many years, it was believed that communication between cells exclusively depends on the release of specific soluble or immobilized mediators and their corresponding receptors. Such a process may involve cell-to-cell contact or the release of mediators into the blood, other bodily fluids (endocrine interactions), or the microenvironment to form gradients (paracrine interactions) [1]. When it was discovered that cells were able to secrete vesicles, it was thought that this was a form of waste elimination. However, it is now known that vesicles represent signaling packages that are able to convey messages to stimulate/inhibit neighboring cells and modify the surrounding microenvironment [2]. The term “rececrine” has been suggested to describe this signaling method, which specifically refers to the secretion of receptors carried by microvesicles (MVs) and their transfer to target cells where they may exert specific functions [3]. There is also increasing evidence for the involvement of MVs in various physiological and pathological events, such as the immune response, cellular differentiation, and vascular and cancer pathologies [4]. 

**Figure 1:**
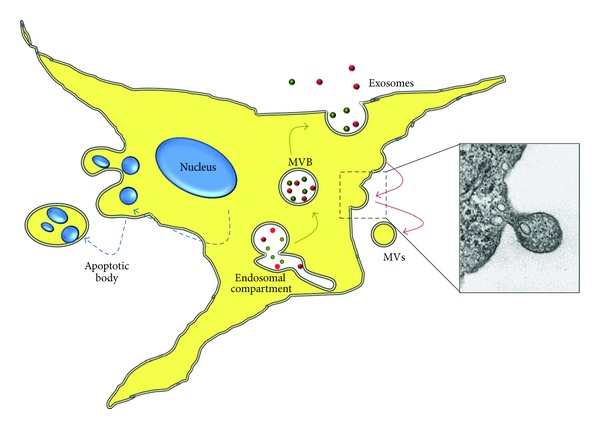
Schematic view of vesicles released from cells. Inset: microvesicle release from human fibroblast plasma membrane (personal original unpublished data).

Cells can release different types of vesicles, the most important of which are apoptotic bodies, exosomes, and shed MVs (Figure 1) [1, 5, 6]; the last two types are primarily involved in the exchange of messages between cells. This paper mainly focuses on the role of MVs as potential clinical biomarkers and also contains a brief overview of all types of vesicles. 

The term “apoptotic bodies” was coined in 1972 [7]. The release of apoptotic bodies from cell membrane is the final consequence of cell fragmentation during apoptosis. Apoptotic bodies have irregular shapes, ranging between 1 and 5 *μ*m in size, and may contain intact organelles and fragmented DNA and histones which, according to Mathivanan et al. [5], are used as unique protein markers to identify these types of vesicles [6]. To date, there is no standard protocol for the isolation of apoptotic bodies [6].

Exosomes, which were first described in 1981 [8], are cup shaped and range from 30 to 100 nm in size. These are produced inside the cell before releasing from multivesicular bodies; they express typical endosomal compartment proteins [2]. However, it is possible that their cup-shaped morphology is the consequence of fixation procedures used for transmission electron microscopy (TEM) analysis [9], as TEM is the gold standard for determining the size of a vesicle. Exosomes are isolated through differential centrifugation followed by sucrose gradient ultracentrifugation, for which their density is between 1.10 and 1.21 g/mL, or through immunoaffinity capture. Typical markers of exosomes include CD63, CD81, CD9, LAMP1, TSG101, Alix, and HSC70 [5]. In addition, exosome membranes are characterized by a low level of phosphatidylserine exposure. Other lipids found in these membranes include cholesterol, ceramide, and sphingomyelin, and lipid rafts are also contained within these membranes [5]. Exosomes have been mainly studied in cancer and immune cells [6]. The ability of exosomes to interact with cells may be due to several potential mechanisms, including direct cellular contact, which is mediated by the interaction of exosomal membranes with target cell receptors, the binding of exosomal membrane proteins released by protease-mediated cleavage to target cell surface receptors, and endocytosis by fusion with target cells [5, 10]. A multitude of pathways may then be activated following cellular interactions with exosomal molecules, including mRNA, microRNA (miRNA), and proteins (e.g., cytoskeletal proteins, heat shock proteins, adhesion molecules, tetraspanins, and proteins involved in signal transduction, transcription regulation, and antigen presentation); induced pathways basically depend on cellular origin of exosomes; exosomes from cancer cells, for example, modulate immune response, stimulate angiogenesis, and are involved in stroma remodeling contributing to tumor progression [5, 11, 12]. 

**Figure 2:**
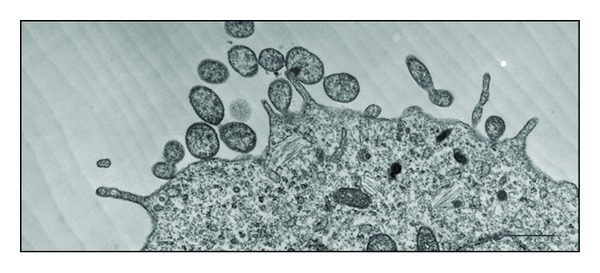
Transmission electron micrograph of the microvesicle shedding process from B16 mouse melanoma cells. Scale bar: 500 nm (personal original unpublished data).

MVs were first described in 1964 [13] and have been intensively studied during the last two decades. These vesicles can have different shapes and range between 100 and 1,000 nm in size, although a low-end size cut-off has not been well established [5, 6]. MVs differ from exosomes, and in addition to their different size variations, the main difference between these types of vesicles is that MVs are formed by the regulated release from outward budding or blebbing of the plasma membrane (inset of Figures 1 and 2). These vesicles may also be isolated by differential centrifugation or capture-based assays [14, 15], and several proteins may be used as MV markers, including flotillin-2, selectins, integrins, CD40, and metalloproteinases [5, 16]; specific marker for MVs has not yet been identified. Moreover, MV membranes are characterized by a high level of exposure of phosphatidylserine, which is translocated from the inner to the outer surface leaflet [17].

Although no standard MV isolation protocols are available, most groups use centrifugation conditions ranging from 18,000 to 100,000 ×g for times ranging from 30 to 60 min [6]. However, it is possible that these conditions pellet mixed vesicle populations because the size distribution of MVs overlaps with that of apoptotic bodies and exosomes at their upper and lower limits, respectively. For this reason, it may be appropriate to combine differential centrifugation with sucrose gradient ultracentrifugation to remove exosomes or to proceed by immunoisolation.

MV cargos include proteins, such as enzymes, growth factors, growth factor receptors, cytokines and chemokines [1], lipids, and nucleic acids, including mRNA, miRNA, ncRNA, and genomic DNA [18, 19]. Various studies of the molecular characterization of MVs have demonstrated similarities and differences with respect to the molecular composition of the cells of origin, suggesting that MVs are not simply miniature parental cells [1, 20]. For example, MVs in human glioma contain a plethora of proteins, cytokines, chemokines, and transcripts that are uniquely contained within vesicles and are undetectable (or expressed in different quantities) in the corresponding parental cells [19].

**Figure 3:**
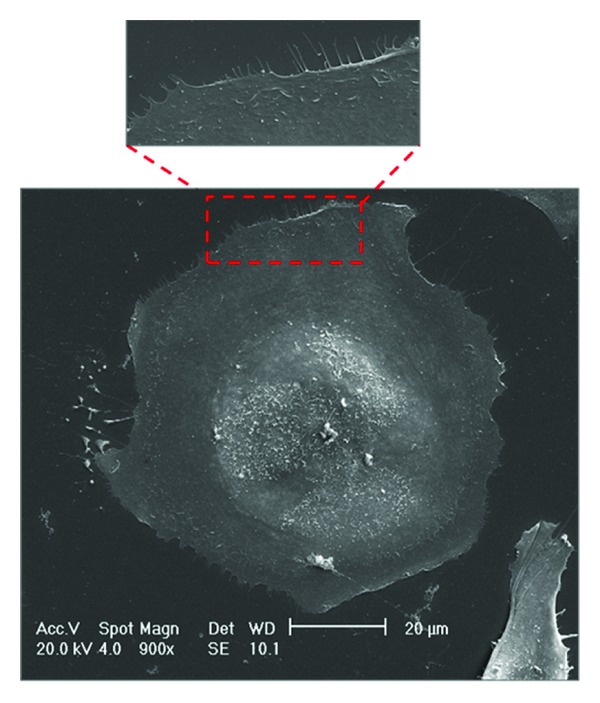
Scanning electron micrograph of human normal ovarian surface epithelium (OSE). The phenomenon of MV shedding is very much reduced in normal cells. Inset: there are no evident microvesicles at the edge of the normal cells (personal original unpublished data).

**Figure 4:**
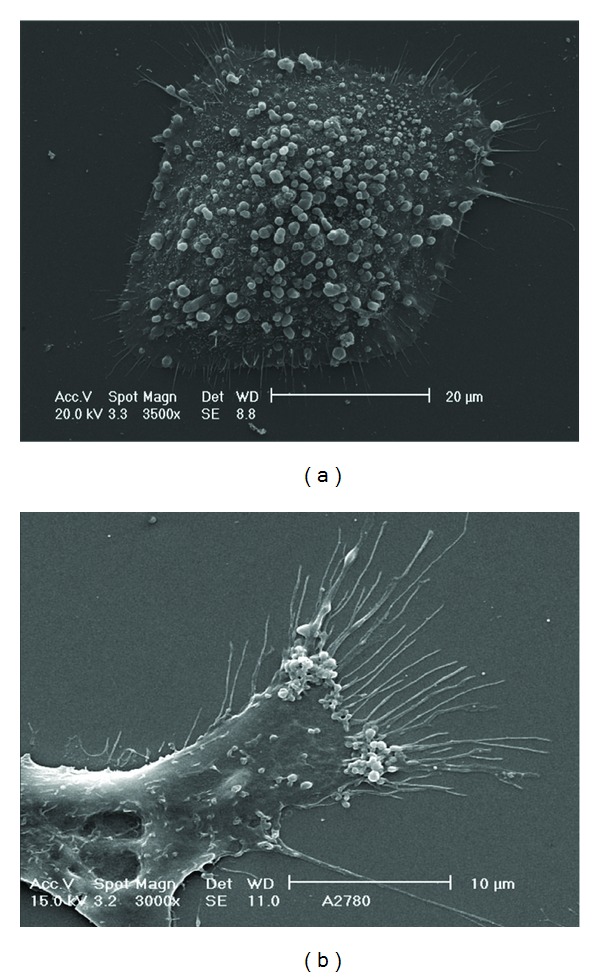
Scanning electron micrograph of OVCA 432 (a) and A2780 (b). It's evident the enormous release of microvesicles with heterogeneous dimensions ranged between 300–100 nm. (a) The microvesicles shedding is visible on whole cell body in OVCA 432; (b) the phenomenon is more evident at the edge of cells (personal original unpublished data).

MVs have been widely studied in several normal cell types, including platelets, red blood cells, and endothelial cells, but have been primarily studied in cancer cells [6, 21, 22]. Importantly, MVs are more easily detectable after the acquisition of a tumorigenic phenotype, as they are shed at low levels in normal and parental cells [23]. In normal cells, indeed, shedding phenomenon occurs in very selected areas of plasma membrane (Figure 3), whereas in tumor cells, a lot of MVs are released from the entire cell surface (Figure 4(a)), especially from invading cellular edges (Figure 4(b)) (personal unpublished original data).

MVs play a role in many aspects of tumor progression, including the following.

- MVs contribute to the progression of cancer cells. The ability of a tumor cell to modify the extracellular matrix is important for enabling tumor progression and invasion, and MVs appear to promote the proteolytic cascade required for the localized degradation of the extracellular matrix through lytic enzymes such as uPA, MMPs, and cathepsins [24]. It has been demonstrated that cancer-derived MVs contain such proteases; for example, prostate carcinoma cell lines release MVs that reach uPA activity levels and are able to adhere to and degrade collagen IV and reconstitute the basal membrane (Matrigel) [25]. Furthermore, MVs from ovarian ascites are rich in MMPs and uPA, the activation of which leads to increased extracellular matrix degradation and facilitates tumor cell invasion and metastasis [26]. Ovarian cancer cell lines release lytic enzymes as well, and the amount and level of proteolytic activity associated with shed vesicles correlate with the *in vitro* invasiveness of cancer cells [23].
- MVs are involved in tumorigenesis too. Indeed, the addition of MVs from PC3 cells (a human prostate cancer cell line with high metastatic potential) to the poorly invasive prostate cancer cell line LnCaP enhanced the adhesive and invasive capabilities of the latter cell type [25].
- MVs help tumor cells evade apoptosis. Some MVs contain caspase 3, which is one of the main apoptotic enzymes. It has been postulated that tumor cells may escape apoptosis by preventing the intracellular accumulation of caspase 3 through the release of MVs containing this enzyme [27]. This hypothesis was confirmed by the observation that cells, if MV release is inhibited, accumulate caspase 3 and undergo apoptosis [28].
- MVs contribute to the induction of transformation. It was demonstrated that glioma cancer cells could transfer through MVs a truncated, oncogenic form of EGFR to glioma cells lacking this receptor and that this transfer was able to transform recipient cells [11]. More recently, it was demonstrated that MVs derived from human cancer cells (i.e., breast carcinoma and glioma cells) may play an important role in oncogenesis, as they were shown to be capable of transforming normal fibroblasts and epithelial cells to adopt the typical cancer cell characteristics (e.g., anchorage-independent growth and enhanced survival capability) through the transfer of the cross-linking enzyme tissue-transglutaminase (tTG) [16].
- MVs promote drug resistance. It was reported that chemoresistant cancer cell lines express more genes related to shedding as compared to chemosensitive cells. Moreover, experiments using the chemotherapeutic agent doxorubicin confirmed the existence of drug accumulation and expulsion through MVs [29], which suggests that MVs released from tumor cells contribute to cellular survival.
- MVs contribute to immunoescape. There are many examples demonstrating how the shedding of MVs mediates interactions between cancer and immune cells to modulate the immune response. MVs released from some cancer cells, such as those of oral cancer, can act as carriers for Fas ligand, which induces apoptosis in T-cells and prevents their cytotoxic effects on tumor cells [30–32]. Moreover, MVs released from human melanoma and colorectal carcinoma cells following fusion with monocytes inhibited differentiation and promoted immunosuppressive cytokine release in the monocytes [32]. Furthermore, some cancer cells (such as squamous cell carcinoma) use MVs to escape from complement-induced lysis; the release of MVs containing CD46, a membrane complement inhibitor cofactor protein, can inactivate complement complexes by inducing the inactivation of C4b and C3b [33].
- MVs promote the induction of angiogenesis. It is well known that tumor growth and survival depends on the formation of new blood vessels that infiltrate the tumor mass [34]. MVs shed from tumor cells may transmit proangiogenic stimuli to endothelial cells through various mechanisms; for example, proangiogenic cargo may be released into the tumor microenvironment or directly transferred to recipient endothelial cells [1]. Some studies have demonstrated that cancer cell MVs can induce the secretion of several proangiogenic factors in stromal fibroblasts to induce endothelial cell proliferation and therefore angiogenesis [35]. It has also been demonstrated that MVs released from tumor cells bearing the EGFR are able to activate the VEGF/VEGFR pathway in endothelial cells [36]. MVs are a rich source of the MMP stimulant EMMPRIN, which is able to promote the angiogenic ability of endothelial cells [37], the proangiogenic growth factor VEGF, FGF-2 [22, 38], and proteases (e.g., uPA, MMPs, and cathepsin B) [15, 22, 25, 39]. Degradation of the basal membrane and extracellular matrix via the actions of lytic enzymes favors angiogenesis and new vessel formation [40]. Moreover, cancer cell-released MVs may contain several molecules (such as sphingomyelin) which could reprogram the endothelial cell response and stimulate their angiogenic ability [41, 42]. Alternatively, cancer-derived MVs taken up by endothelial cells can turn on VEGF production, inducing autocrine stimulation [36].

In summary, it is clear that MVs are able to directly and indirectly modulate the behavior of surrounding cells through their delivery of proteins and nucleic acids. Moreover, the effects that MVs have on target cells have been extensively studied, although it remains unclear how MVs interact with target cells, that is, whether they fuse with the plasma membrane or are taken up by endocytosis. 

## 2. MV Isolation from Biological Fluids

The quantity and molecular characteristics of circulating MVs reflect not only their cellular origin but also the stimulus that triggered their release. Thus, the isolation and analysis of circulating MVs, which are released into bodily fluids exposed to primary tumors (e.g., blood, urine, saliva, ascites, pleural effusion, and spinal fluid), may provide the opportunity to assess pathological and cancer-related biological information. Furthermore, this type of analysis may enable rapid and repeated evaluation without the need for invasive procedures such as surgical biopsy, which can be affected by sampling error [1, 18, 43]. MVs have also been studied to identify a potential association with the prognosis of several pathologies, including thrombosis, sepsis, coronary artery syndrome, multiple sclerosis, and some cancer types [44–48]. 

 As a result of studies in which it was assessed the half-life in the bloodstream of labeled MVs, it has been hypothesized that MVs have a lifespan of about 15–60 min in the blood circulation [49, 50]; the rapid elimination could be because they are rapidly taken up by recipient cells. It is also possible that other forms of bioelimination may exist, for example, due to the interaction of phosphatidylserine exposed on their surface with phagocytic system. Nevertheless, it is not possible to exclude the possibility that biological activities of MVs may persist long enough (some days perhaps) giving the chance to perform desired analysis [1]. 

To date, there are no validated methods for the isolation, identification, characterization, or detection/quantification of circulating MVs. Moreover, it must be noted that the presence of MVs derived from nontumor cells in bodily fluids may be a further complicating factor that requires the development of strategies enabling selectively isolate tumor-derived MVs, which may represent a relatively small fraction of the total number of isolated MVs [18]. The lack of adequate validation methods greatly limits the potential use of MVs as clinical markers, although several studies have been conducted to assess the reliability of this approach [43, 51–54]. 

It appears to be important to standardize the preanalytical procedures in order to study biological fluids, as centrifugation procedures or the choice of a specific anticoagulant may affect the reproducibility of MV analyses. Time and storage temperature may also be critical parameters, although the freezing of plasma (useful for large scale analysis) for more than one year was shown to minimally affect the recovery of MVs [43]. Two main strategies have been proposed for the isolation of MVs, and these include techniques based on MV physical properties and those based on MV biochemical features. In the former approach, size and density are used as reference parameters, and serial centrifugations and flotation in sucrose gradients, which is occasionally combined with size-exclusion chromatography, are mainly used, although size discrimination based on dielectrophoresis sorting has been employed as well. In the latter approach, magnetophoretic sorting or immunoaffinity chromatography are used [18].

Cytometry is the most widely used method to detect and quantify MVs in biological fluids because it uses both size and affinity measurements (through conjugation with specific fluorescent antibodies). The number of MVs is important because these numbers seem to correlate with various pathologies and nonphysiological conditions and may aid in the diagnosis and determination of prognosis of these conditions. However, it must be noted that vesicles smaller than 200 nm cannot be distinguished from instrumental noise; thus, exosomes and smaller MVs cannot be detected using this technique. Nevertheless, there have been many studies that have standardized and improved MV analysis through the use of flow cytometry [6, 55, 56]. More recently, a novel strategy based on the differential light scattering of different size particles solved in a fluid medium (NanoSight) has been used to detect and quantify MVs [18].

Initially, annexin V was used as a marker for MVs; however, evidence for a substantial proportion of annexin V-negative MVs was found [6, 57]. Thus, alternative labeling was proposed based on the cellular source of the MVs [4, 58, 59].

## 3. MVs as Cancer Biomarkers

A biomarker, or biological marker, is a substance whose detection is used as an indicator of a biological state and whose changes are correlated with the progression or the response of the disease to a given therapeutic treatment. Ideally, a biomarker should be specifically associated with a particular disease. Consequently, it should be able to discriminate between two pathological or physiological conditions even if they are similar. It would also be convenient if the biomarker could be identified in a biological sample that is easily obtainable, for example, blood, urine, or saliva. Moreover, biomarker expression levels should be able to predict aspects of the corresponding pathological/physiological condition. Moreover, for routine use, noninvasive detection methods that are accurate, fast, and potentially inexpensive should exist [60]. 

As previously mentioned, many studies have been conducted to better understand the role of circulating MVs in various clinical conditions. The best characterized MVs are those derived from platelets and endothelial cells, and their alterations (mainly elevated levels) are involved in numerous clinical disorders such as cardiovascular diseases (e.g., hypertension, atherosclerosis, and congestive heart failure) [61–63], autoimmune diseases (e.g., rheumatoid arthritis, vasculitis, type I diabetes mellitus, and multiple sclerosis) [64–67], and hematological and cerebrovascular diseases [68, 69]. 

 However, in recent years, tumor MVs have gained attention as potential biomarkers because tumor cells are able to constitutively release large amounts of MVs bearing tumor-specific antigens into the bloodstream and other bodily fluids. For example, solid tumors that are difficult to reach and detect may reveal their presence by releasing MVs, and the presence of tumor-derived MVs in biological fluids may also be useful for detecting metastases [70]. Moreover, in addition to protein antigens, MVs are able to carry RNAs, particularly miRNAs. miRNAs and other molecular features of MVs represent a unique combination representative of the cancer cells from which they were derived [20]; thus, their presence in cancer-derived MVs may serve as a novel source of disease-related information and possibly as unique, specific, and identifiable cancer biomarkers that may prove useful for screening and diagnosis [1]. Tumor-specific markers, such as mucin in adenocarcinoma, may also be used in the early detection of cancers [27].

 MVs have been detected in the circulation of patients with several cancers, such as breast, ovarian, lung, prostate, colorectal, and gastric cancers [27]. In gastric cancer, MVs are notably increased in patients with stage IV disease. MV levels are also elevated in cancers with associated thromboses, such as colorectal carcinoma, breast cancer, and pancreatic adenocarcinoma [71, 72]. In patients with bladder cancer, MVs were isolated from urine and were identified eight proteins whose levels were elevated with respect to healthy controls, which indicated that the protein composition of urine MVs could be used for the early detection of this pathology [73]. MVs from patients with glioblastoma demonstrated high levels of CD133 and the transcript encoding the oncogenic form of EGFRvIII. Furthermore, it is intriguing that tumor removal correlates with the disappearance of circulating MVs [19, 74] and that MVs may maintain proteins with the same functional state (e.g., phosphorylation) as those typical of their parental cancer cells. This property may be potentially utilized to follow the effects of some anticancer drugs [40].

 Some studies have been conducted to assess the use of MVs in prognosis too; in patients with disseminated breast and pancreatic cancer with higher levels of TF (Tissue Factor) and MUC1 (epithelial mucin) in MVs was shown a lower survival rate at 3–9 months followup compared to those with low TF-activity and no MUC1 expression [71]. In patients with hormone-refractory prostate cancer, platelet MVs levels were predictive of outcome; overall survival was significantly shorter in those patients with MVs level above the cut-off compared to those patients whose level was below it [75]. Patients with gastric cancer at stage IV showed higher levels of MVs compared to controls, and plasma levels might be useful to predict metastasis formation [72].

 In the future, the use of MVs as serum biomarkers may facilitate cancer diagnosis in controversial cases and help to avoid the use of invasive procedures, primarily those involving surgical biopsies of organs in which repeated biopsies are unrealistic (e.g., the pancreas, ovaries, or central nervous system) [18]. It has been hypothesized that because the molecular profiles of cancer cells change with disease progression, MVs may be useful for disease staging or even to evaluate the response to therapy by permitting an accurate assessment of a patient's responsiveness and personalization of treatment [18]. The analysis of MVs may also be used to detect tumor recurrence [18, 70]. Moreover, if we assume that MVs are representative of the molecular features of the parental cancer cells, their profiling may be useful for creating targeted and personalized anticancer therapies. For example, in some tumors, including ovarian, breast, and gastric cancers, the level of the HER-2/neu oncogenic receptor was elevated, and the protein was detected in MVs in the serum, which suggests that these patients may benefit from current therapeutic treatments targeting HER-2 [18, 76].

 Although the results presented to date are undoubtedly promising, further investigation is required to determine the feasibility of the use of MVs as circulating cancer biomarkers. Furthermore, the routine use of MVs in diagnosis and prognosis requires some additional precautionary notes. First, the development of sensible instruments is needed to be able to isolate all of the MVs from an analyzed sample (e.g., the blood or other bodily fluids exposed to tumors). Second, because samples will contain MVs derived from nontumor cells, advanced strategies with greater specificity are needed to target and isolate pathological MVs that may be diluted in the biological sample.

## 4. MV-Associated miRNAs as Possible Biomarkers for Human Ovarian Cancer

Ovarian cancers comprise a heterogeneous group of neoplasias that are mostly epithelial cancers characterized by mucinous, serous, endometrioid, and clear cell subtypes and are derived from ovarian surface epithelium or inclusion cysts. However, these cancers also include sarcomas and sex-cord stromal, germ cell, and mixed tumors, which may be rare [77]. Ovarian cancer is the most lethal gynecologic malignancy and is characterized by poor prognosis with an overall 5-year survival rate of approximately 50%. If the cancer is diagnosed while confined to the ovary, the 5-year survival rate could become 90%, but this occurs only in a small percentage of patients (approximately 20%) [78, 79]. Ovarian cancer can be identified at the following four stages: stage I, the cancer is limited to one or both ovaries; stage II, the cancer is present in one or both ovaries as well as in pelvic extensions or implants; stage III, peritoneal implants are present outside of the pelvis or are limited to the pelvis with an extension to the small bowel or omentum, and there may also be metastasis on the liver surface; stage IV, distant metastases to the parenchymal compartment of the liver or outside of the peritoneal cavity are present [80].

The ovarian cancer diagnosis is often delayed because the first specific symptoms, which are mainly related to the presence of large tumors or extensive ascites, appear only during an advanced disease stage [81–83]. However, early diagnosis is fundamental for offering patients a better chance of being cured using available therapies, such as surgery or, in some cases, chemotherapy with the combination of platinum and taxane. The more a tumoral mass is reduced by surgery, the more often the following chemotherapy is effective [84]. Unfortunately, tumor recurrence frequently occurs, and patients can develop resistance to additional therapies [79]. 

Currently, imaging methods such as computer tomography-positron emission tomography (CT-PET), fluorodeoxyglucose-PET (FDG-PET), magnetic resonance, transvaginal and transabdominal sonography, and the serum marker CA 125 are used as diagnostic tools [79]. CA 125 is undoubtedly the most carefully studied and extensively used biomarker despite being characterized as having low sensitivity and specificity [85]. Many gynecologic and nongynecologic pathological conditions showed increased serum levels for this marker such as endometriosis and adenomyosis, pelvic, peritoneal, pleural, and musculoskeletal inflammatory diseases, hepatitis, and pancreatitis [81, 86]. In addition, physiological conditions such as menstruation or pregnancy can be associated with elevated CA 125 levels [81], and it should be noted that the CA 125 level remains normal in some women with ovarian cancer [81].

Biomarker specificity is fundamental to be sure that the patient really has this specific pathology, because a definitive diagnosis often requires abdominal surgery; thus, there can be a great negative impact on women who have false-positive results [87]. CA 125 remains the most effective biomarker despite studies that have searched for alternative and potentially useful serum biomarkers, including CA 19-9, CA 15-3, CA 72-4, CEA, HE4, lysophosphatidic acid (LPA), Haptoglobin-*α* (HP-*α*), Bikunin, and OVX1 [81, 87]. With the exception of HE4, which appears to have high sensitivity even at early stages, all of these markers have shown disadvantages, such as poor correlation with the clinical course or low specificity [81]. In fact, no other ovarian cancer serum marker appears to be able to replace or complement CA 125, which highlights the need to find a novel marker for this disease. Furthermore, the discovery of alternative serum biomarkers for early diagnosis is vitally important.

One new insight into ovarian cancer biomarker identification occurred after the discovery of miRNAs. miRNAs are small (19–25 nucleotides), single-stranded, noncoding RNAs that are responsible for gene expression regulation at the posttranscriptional level. In animals, miRNAs act by inhibiting mRNA translation at the initiation or elongation step, which blocks the translation of mRNAs from several important genes into corresponding proteins [88]. Their regulatory functions mainly affect cell proliferation and differentiation and cell cycle regulation [89]. It has been widely shown that abnormal miRNA levels are associated with many pathologies, including cardiovascular disease, diabetes, rheumatoid arthritis, and cancer [90]. The role of miRNAs in cancer has been discussed in several studies, and a substantial number of miRNAs, which normally act as tumor suppressors, are downregulated in cancer cells. In contrast, some miRNAs that normally act as oncogenes are expressed at higher levels in cancer cells. The consequences of these changes in miRNA levels include the altered expression of target oncogenes and tumor suppressor genes, which are undoubtedly involved in carcinogenesis [81].

In several cancers, including ovarian cancer, it has been demonstrated that the expression of a specific subset of miRNAs may potentially be used in clinical practice, for example, for screening or early diagnosis to evaluate the response to therapeutic treatments [91, 92]. It was also demonstrated that miRNA profiles can be used to distinguish between various histological ovarian cancer subtypes [93], and some profiles also appear to be closely related to early relapse in patients with advanced-stage tumors [94]. Furthermore, some miRNAs are consistently and significantly overexpressed in ovarian cancer, including miRNAs belonging to the miR-200 family (i.e., miR-200a, miR-200c, and miR-200b), whereas miRNAs of the let-7 family, miR-140, miR-145, and miR-125b1 are consistently downregulated in ovarian cancer. Altered expression has also been reported for other miRNAs, such as miR-21, miR-99a, miR-125b, and miR-199a [78, 93, 95] (Table 1). Moreover, a correlation between miRNA features and chemoresponse was also reported in other cancers, including leukemia, colorectal adenocarcinoma, and breast, pancreatic, and lung cancers, which indicates the potential use of miRNAs for diagnosis and predicting patient survival rates and risk of recurrence [78, 96–101]. It is interesting to note that miRNAs can be detected in the bloodstream; however, for stable expression, miRNAs must be protected from RNases, which are abundant in the blood and are able to degrade approximately 99% of RNA species within 15 min [102]. Thus, it is not surprising that miRNAs in serum are contained in apoptotic bodies, exosomes, and MVs [81]. The association between miRNA profiles and cancer type and stage, as well as the stability of miRNAs in the blood and other biological fluids, makes them hypothetically useful markers for early cancer diagnosis. These findings can be applied to ovarian cancer as well, as it was demonstrated that exosome-associated miRNAs may serve as novel serum diagnostic biomarkers [103]. It was convincingly demonstrated that the miRNA signatures of exosomes released from tumors in the bloodstream were distinct from those observed in patients with benign disease and could be strongly correlated with the ovarian cancer stage of the patient. The level of detectable miRNA is significantly increased in women with invasive ovarian cancer compared to healthy patients or women with benign ovarian cancer [104, 105]. Also, the levels of tumor-derived exosomes in the bloodstream increase with increasing disease stage [105].

It should also be noted that MVs released from ovarian cancer cells may be present in biological fluids, like exosomes. Some years ago, it was demonstrated that ovarian cancer cells are able to release a large amount of MVs *in vivo* [106]. In addition, a study conducted on biological fluids obtained from patients with gynecological diseases demonstrated that benign and tumor fluids contained MVs, but that malignant tumor fluids were found to have a larger quantity of vesicles than fluids from nonmalignant pathologies (e.g., ovarian serous cysts, mucinous cystoadenomas, and fibromas). Moreover, tumor progression has been shown to correlate with an increase in MVs abundance in ascitic fluids. Importantly, increases in MVs levels appear to occur several months prior to elevation of CA 125 in serum, which further suggests that MVs have the potential to serve as early biomarkers [106]. In addition, it should be highlighted that the miRNA features of ovarian cancer-derived MVs may be useful as well, as the analysis of such MVs demonstrates distinct miRNA signatures associated with ovarian cancer (our unpublished data).

## 5. Conclusion

To date, very few molecules, particularly CA 125, are used as routine ovarian tumor markers. For this reason, many novel serum biomarkers are under investigation for use as diagnostic and prognostic tools to evaluate the therapeutic treatment response. Because cancer cells may release MVs into the bloodstream that contain similar miRNA characteristics as the cells from which they originated, miRNA signatures appear to be promising tools for the ovarian cancer field. It has also become evident that MVs may represent an ideal biomarker for ovarian cancer diagnosis and prognosis. However, additional ovarian cancer-derived MV characteristics should be evaluated to confirm this intriguing hypothesis. Furthermore, it is necessary to develop the ability to isolate and quantify tumor derived-MVs from the blood and other biological fluids.

## Figures and Tables

**Table 1:** A list of miRNAs with altered expression in ovarian cancer.

miRNAs altered	References mentioning upregulation	References mentioning downregulation
let-7a/b/c/d/e/f		[101, 107–109]
miR-10a		[109]
miR-10b		[110]
miR-16	[110]	
miR-20a	[110]	
miR-21	[105, 109, 110]	[107]
miR-23a/b	[110]	
miR-26a		[108, 110]
miR-26b	[108]	
miR-27a	[110]	
miR-29a	[101, 105, 109]	[110]
miR-29c	[107, 109]	
miR-92	[105]	
miR-93	[105]	
miR-99a	[107, 108]	[110]
miR-103	[108, 109]	
miR-106b	[109]	[107]
miR-122		[107]
miR-125a		[110]
miR-125b	[109, 111]	[101]
miR-125b1	[93]	
miR-126	[105]	
miR-127		[105, 108]
miR-130a		[111]
miR-134		[107, 108]
miR-140		[93]
miR-141	[109, 110]	[107]
miR-143		[109]
miR-145		[93, 109, 110]
miR-146b	[109]	
miR-155		[107]
miR-182	[108, 109]	
miR-199a	[101, 107]	[93]
miR-200a/b/c	[93, 95, 101, 109, 110]	
miR-214	[101]	[110]
miR-221	[107]	
miR-222		[95, 108]
miR-296	[107]	
miR-302d	[101]	
miR-320	[101]	
miR-335		[111]
miR-346		[107]
miR-410		[108]
miR-422a	[109]	[107]
miR-424	[101]	
miR-432		[108]
miR-494	[107]	[101]
miR-508	[107]	[109]
miR-519a		[107]
miR-648		[107]
miR-662		[107]
miR-663	[107]	

## Abbreviations

CEA: Carcino-embryonicantigen
CT-PET: Computertomography-positronemissiontomography
EGFR: Epidermalgrowthfactorreceptor
EMMPRIN: Extracellularmatrixmetalloproteinaseinducer
FDG-PET: Fluorodeoxyglucose-positronemissiontomography
FGF-2: Fibroblastgrowthfactor-2
HE4: Humanepididymisprotein4
HER-2: Humanepidermalgrowthfactorreceptor2
HP-*α*: Haptoglobin-*α*
HSC70: Heatshockcognate71 kDaprotein
LAMP-1: Lysosomal-associatedmembraneprotein1
LPA: Lysophosphatidicacid
miRNA: MicroRNA
MMPs: Matrixmetalloproteinases
MVs: Microvesicles
ncRNA: NoncodingRNA
TEM: Transmissionelectronmicroscopy
TSG101: Tumorsusceptibilitygene101
tTG: Tissue-transglutaminase
uPA: Urokinase-typeplasminogenactivator
VEGF: Vascularendothelialgrowthfactor
VEGFR: Vascularendothelialgrowthfactorreceptor.
